# An Internet-Based Intervention (Condom-Him) to Increase Condom Use Among HIV-Positive Men Who Have Sex With Men: Protocol for a Randomized Controlled Trial

**DOI:** 10.2196/resprot.2723

**Published:** 2013-10-13

**Authors:** Joyal Miranda, José Côté, Gaston Godin, Martin Blais, Joanne Otis, Yann-Gaël Guéhéneuc, Ghayas Fadel, Luisa Barton, Shawn Fowler

**Affiliations:** ^1^Faculty of Community ServicesDaphne Cockwell School of NursingRyerson UniversityToronto, ONCanada; ^2^Faculté des Sciences InfirmièresUniversité de MontréalMontréal, QCCanada; ^3^Centre de Recherche Centre Hospitalier de l’Université de MontréalMontréal, QCCanada; ^4^Faculty of NursingUniversité LavalLaval, QCCanada; ^5^Faculté des Sciences HumainesDépartement de SexologieUniversité du Québec à MontréalMontreal, QCCanada; ^6^Département de Génie Informatique et Génie LogicielÉcole Polytechnique de MontréalMontreal, QCCanada; ^7^Coalition des Organismes Communautaires Québécois de Lutte Contre le SidaMontreal, QCCanada; ^8^Hassle Free ClinicToronto, ONCanada

**Keywords:** HIV-positive, men having sex with men, condom use, self-efficacy, intention, HIV prevention, pilot study, intervention

## Abstract

**Background:**

In the recent years, the Internet has been used as a medium to find sexual partners and engage in risky sexual behavior. This has changed the way in which men having have sex with men (MSM) seek sexual partners and has increased the number of high-risk sexual encounters. Therefore, developers of human immunodeficiency virus (HIV)-prevention interventions have also started using the Internet as a viable medium to promote safe sexual behaviors. However, much of the efforts thus far have been aimed at HIV-negative rather than HIV-positive MSM. HIV-positive individuals continue to engage in risky sexual behaviors and thus constitute an important group in which HIV prevention strategies need to be addressed. Therefore, HIV prevention in HIV-positive MSM is a critical issue.

**Objective:**

Condom-Him, an Internet-based intervention tailored to increase condom use among HIV-positive MSM, was developed with the aim of improving condom use, self-efficacy, and intentions to use condoms among these individuals. The acceptability and feasibility of this Internet-based intervention will be examined in a pilot study.

**Methods:**

We will perform a randomized controlled parallel-group superiority trial. HIV-positive MSM who currently engage in unprotected anal sex will be recruited for the study. Participants will be randomly assigned using a one-to-one allocation ratio generated by the computer program. The researchers will be blinded to participant’s group assignment. Participants will be assigned either to use the Condom-Him intervention (experimental arm) or to view a list of websites containing HIV/AIDS related information (control arm). Self-administered questionnaires will be provided online before randomization (baseline) and two weeks after intervention (post-test).

**Results:**

The study will include a total of 60 participants with 30 in each group. The results from this pilot study will provide further evidence for a larger study to examine the effectiveness of this intervention and will provide a cost-effective and widely accessible approach to HIV prevention for HIV-positive MSM.

**Conclusions:**

Internet-based interventions for HIV-positive MSM, a population that has been under-represented in the efforts for positive prevention of HIV within Canada, have the potential to provide a cost-effective strategy, which influences the way in which information is accessed and provided to high-risk individuals. The advantages of an Internet-based intervention include the potential to provide consistency in the delivery of an intervention and the ability to disseminate the intervention to a wider population. Internet-based interventions are perceived as vital tools in combating HIV infection within the realm of social media. Therefore, it is important to determine the feasibility and acceptability of these interventions before implementing them.

**Trial Registration:**

Clinicaltrials.gov: NCT01726153; http://clinicaltrials.gov/ct2/show/NCT01726153 (Archived by WebCite at http://www.webcitation.org/6Jljzip8B).

## Introduction

### Overview

The number of human immunodeficiency virus (HIV)-positive individuals continues to increase in Canada. Among the high-risk groups, the group of men having sex with men (MSM) accounted for the greatest proportion (44%) of new infections in 2008 [1]. Various studies, which target the sexual risk behaviors of HIV-positive MSM, have found that 10% to 60% of these individuals do not routinely practice safe sex [2]. In addition, MSM recently infected with HIV had a median of 20 sexual partners within the previous year, and they continued to repeatedly engage in high-risk sexual behavior, particularly unprotected anal intercourse [3]. Moreover, 34% of MSM recently diagnosed with an HIV infection did not change their risk behaviors after the diagnosis of their condition, and 20% actually increased their risk behaviors after the diagnosis [3]. The potential outcomes of engaging in unprotected sex with partners who are HIV-negative or whose status is unknown are the possibility of infecting others with HIV and putting oneself at risk for contracting sexually transmitted infections (eg, syphilis, gonorrhea, and herpes virus infection) [4]. Many individuals who engage in risky sexual behaviors use the Internet to meet their sexual partners, and the Internet itself may facilitate such risk-taking behaviors [5,6]. In keeping with the current trends, the individuals working in the field of prevention of HIV infection need to incorporate the same medium as that used by high-risk individuals to reduce risky sexual behaviors. Thus, developing technological innovations such as Internet-based interventions is critical.

### Computer-Based Online Interventions

Computer-technology-based interventions (CBIs) [7] provide an alternative to human-delivered interventions for prevention of HIV infection. Noar, Black, and Pierce [8] defined CBIs as those that use computer technology as the primary or sole medium for intervention delivery. To date, few trials of health interventions that use computer technology and are conducted entirely online have been performed [9]. In the case of such interventions, all aspects of the study must be implemented online, that is, participant recruitment, randomization process, data collection, and delivery of the actual intervention. Many of the current CBIs recruit participants using methods other than the Internet and collect data in a face-to-face format, and only provide the actual intervention online.

### Advantages of Internet-Based Studies

Previously, HIV prevention interventions were mainly delivered on individual or group levels. However, many clinical and community settings do not have adequate human resources required for implementation of such methods [8]. Therefore, the ability to disseminate efficacious interventions into many practice settings remains limited. In addition, the barriers and challenges of intervention fidelity have limited the public health impact of behavioral HIV prevention interventions [8]. CBIs have the advantage of providing consistency in the delivery of an intervention and enable participants to initialize their participation in the study at any time of the day and at a location convenient to them. Online participation removes barriers such as scheduling times and human resources that may restrict participants’ participation or the ability to provide the intervention to a number of individuals. For example, individuals in rural settings, for whom it may be difficult to access the resources, would able to participate in the CBIs without the costs associated with travel and/or taking time off from work. Further, the anonymity provided by Internet-based interventions is an advantage, specifically to those who may feel stigmatized. For instance, individuals who have not yet disclosed their HIV infection status and who may feel hesitant about participating in an intervention geared towards those who are HIV-positive could participate in such CBIs because the disclosure factor would not be relevant with an Internet-based intervention. In addition, a previous study has shown that typically, participants answering questionnaires online report higher rates of sexual behavior than in pen-and-paper or interview formats [7]. Moreover, the cost associated with Internet-based interventions is low, and human facilitators are not required for implementation of the intervention; therefore, the intervention can be run on numerous occasions. In addition, the data collection methods are less costly because questionnaires do not have to be mailed to the participants, and in-person are not required for collecting data [9]. Finally, online data collection is quicker, because the need for booking appointments or mailing questionnaires is eliminated. Online questionnaires are immediately available to participants for completion, and thus provide immediate accessibility to the data.

### Interventions to Foster Condom Use Among HIV-Positive MSM

Previously, HIV prevention programs were primarily focused on HIV-negative subjects or subjects whose HIV status was unknown. Safe sex behaviors such as condom use should also be practiced by HIV-positive individuals. However, little attention has been focused on interventions targeting this population. The National Institute of Health and the Centers for Disease Control and Prevention have identified a need for behavioral interventions in HIV-positive individuals [10]. To fill this scientific gap, we plan to conduct an online pilot randomized controlled trial (RCT) to evaluate the acceptability and feasibility of Condom-Him, an Internet-based intervention developed to promote condom use among HIV-positive MSM with partners who are HIV-negative or whose status is unknown.

The primary outcome of the tailored Internet-based intervention is to increase condom use among HIV-positive MSM. The primary objective of this study is to examine the overall acceptability and feasibility of the tailored Internet-based intervention. The secondary objective of this study is to examine the preliminary efficacy of the tailored Internet-based intervention in increasing self-efficacy and intention to use condoms.

## Methods

### Study Design

**Figure 1:**
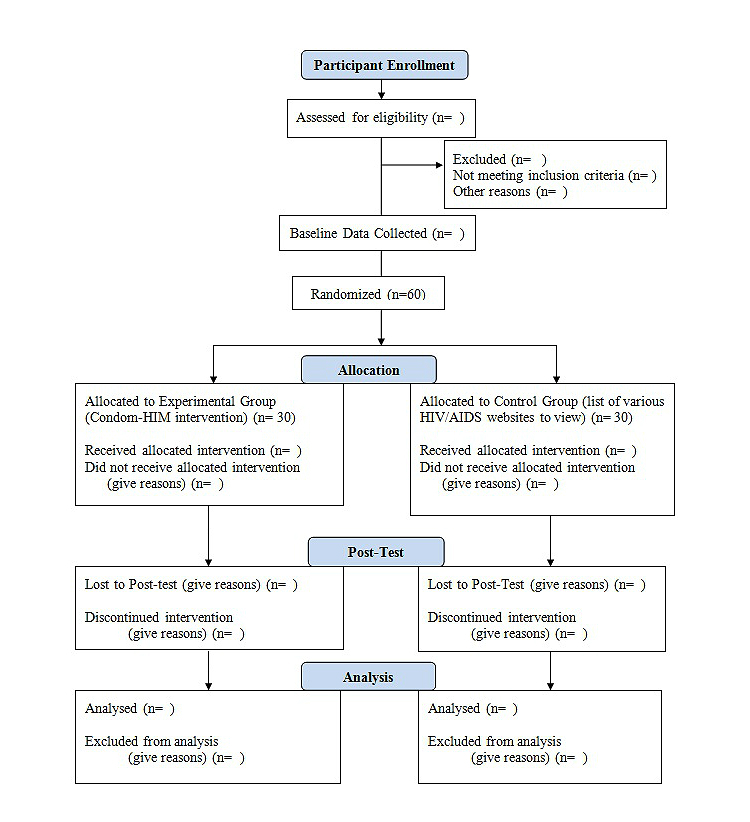
Flow of participants through randomized control trial. As the study is in progress, some n='s are left blank.

The design of the proposed pilot study to evaluate the acceptability and feasibility and acceptability of the tailored Internet-based intervention in increasing condom use among HIV-positive MSM is a randomized controlled parallel-group superiority trial (NCT01726153). The flow of participants within the randomized control trial is shown in Figure 1. Participants will be randomly assigned using a one-to-one allocation ratio. Random assignment will occur only after participants have completed baseline questionnaires. The random assignment will be performed by the computer system that has been programmed by the computer/Web-intervention programmer. Participants cannot be blinded to their assigned arm. Individuals within the control arm receiving a list of various websites will be aware they have not been allocated to use the tailored Internet-based intervention. Participants in the experimental arm (Condom-Him) will be aware that they have been allocated to use the tailored Internet-based intervention. The researcher will be blinded to the participant’s treatment assignment. The research assistant, who will be downloading the data, will be the only individual aware of the participant’s assignment. The research assistant will not have any participation in the random assignment of participants because this process does not involve any individuals and is entirely performed using the computer program. The outcomes of the study will be collected at two time points: pre-intervention (T0) and two weeks after intervention (T1). The participants will be sent an email two weeks after the intervention to remind them to revisit the study website to complete the post-test questionnaires. The study has been approved by the research ethics board of both the academic and research institutions.

### Experimental Arm: Condom-Him

**Figure 2:**
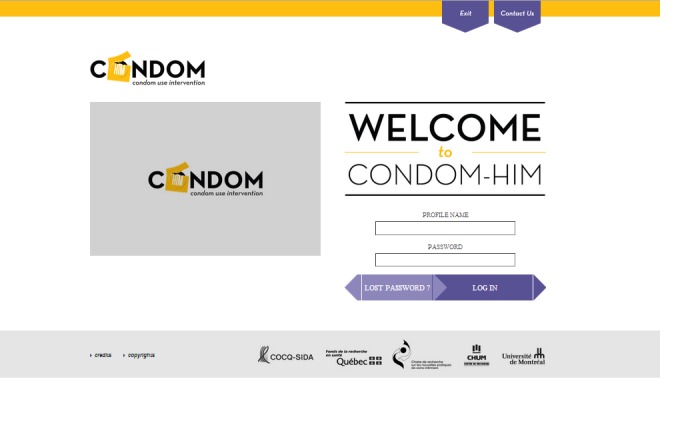
A screenshot of the Condom-Him intervention study.

The tailored Internet-based intervention Condom-Him was designed to increase condom use, self-efficacy in condom use, and intention to use condoms among HIV-positive MSM and their partners who are HIV-negative or whose HIV infection status is unknown. The welcome page of the Condom-Him intervention study is shown in Figure 2. Condom-Him is a tailored interactive single session lasting approximately one hour. The single session is tailored to the specific needs of the participants determined from their responses to the baseline questionnaire about self-efficacy and intention to use condoms. The intervention was tailored on the basis of cutoff scores for self-efficacy and intention measures. On the basis of their responses, the participants are classified into the high or low self-efficacy in condom use groups. A cutoff score of 0-23 indicates high self-efficacy, and a cutoff score of 24-48 indicates low self-efficacy. In addition, the intention to use condoms was also tailored using a cutoff score of 0-2, which indicated a low intention, and a score of 3, which indicated a high intention to use condoms. Subsequently, the participants were profiled into one of the four possible profiles: (1) high self-efficacy and intention, (2) low self-efficacy and intention, (3) low self-efficacy and high intention, and (4) high self-efficacy and low intention. The participants were then guided through the online session by a virtual “peer”, who provides tailored intervention messages particular to their profile in addition to providing stories and videos and interactive activities to increase self-efficacy and intention to use condoms. The single session is divided into three segments to increase condom use, self-efficacy in condom use, and intention to use condoms as follows: (1) planning condom use when having anal intercourse, (2) negotiating the use of a condom with a partner, and (3) choosing not to have sexual intercourse without a condom.

Within each of the three segments of the intervention, participants are given tailored messages pertaining to the focus of the segment. The Intervention Mapping method was used to develop the intervention. This process consists of six consecutive steps to systematically develop health promotion programs using theory, empirical evidence from the literature, and additional evidence from research [11]. The theory-informed methods and practical strategies selected to increase condom use practices of the target population were consistent with the social cognitive theory and the theory of planned behavior.

### Control Arm

The control arm involves links to various websites that address standard information HIV/acquired immunodeficiency syndrome (AIDS). Participants in the control group are invited to view this list of predetermined websites at their convenience.

### Sample Size

Because of the pre-experimental nature of the proposed pilot RCT study, we aim to achieve a sample size of 60 with 30 participants per group. The sample size calculation is based on the rule of 10 cases per independent variable included in the analysis [12]. A convenience sample of participants will be randomly assigned either to the experimental arm (Condom-Him) or to the control arm, which includes a list of various websites providing information relating to HIV/AIDS.

### Planned Inclusion Criteria

Individuals will be selected to participate in the study if they meet the following preset inclusion criteria: (1) 18 years of age or older, (2) HIV-positive, (3) MSM, (4) engage in unprotected anal intercourse with a partner who is HIV-negative or whose HIV infection status is unknown, (5) read English, and (6) have access to a computer and the Internet.

### Participant Recruitment

A two-pronged approach will be used to recruit participants. The first method of recruitment will use Internet-based methods (ie, advertisement in chat rooms, online classified advertisements, social media such as Facebook and Twitter, and links to the intervention website posted within various HIV/AIDS community center websites). The second method of recruitment will use offline strategies (ie, flyers and brochures will be posted in various community sexual health clinics). In addition, advertisements in local newspapers will be used to aid in participant recruitment. Participants interested in the study will be directed to the study website where further information about the study will be provided via a video. The interested participants will be asked to complete a Web-based consent form. The consent form will provide information relating to the study in addition to telephone numbers and email addresses of the research staff for further questions before consenting to participate.

### Random Assignment

Eligible consenting participants will be randomly assigned either to an experimental group (Condom-Him) or to a control group (list of websites with various HIV/AIDS information). The participant assignment protocol will be based on a ratio of 1:1, which will be performed using a computer program. The computer program will automatically allocate participants randomly to the experimental or control groups. Because of the automatic nature of the allocation process, direct exposure to the treatment allocation process by any members of the research team is eliminated.

### Measurements

All measurements are self-administered online. The data that is collected online will be stored on a secure server located at the Research Chair, Centre Hospitalier de l’Université de Montreal.

### Sociodemographic Characteristics

Standard questions will be used to record age, level of education, and sexual history such as date of HIV-positive result, current involvement in treatment, current viral load count, type of partner (casual/primary partner), perceived status of the partner (positive, negative, or unknown), and previous exposure to any sexually transmitted diseases.

### Self-Efficacy

The self-efficacy for condom use measure contains a set of items to rate the belief of participants that within the next 6 months, they will be able to use condoms every time they have anal sex in different situations with a partner who is HIV-negative or whose HIV infection status is unknown. A 4-point response scale, which ranges from “strongly agree” (0) to “strongly disagree” (4) is used. The psychometric properties of the measure show good internal consistency; Cronbach alpha=.96 [13].

### Intention to Use Condoms

To measure the intention of the participants to use condoms, participants are asked three questions about their intentions over the next 6 months to use a condom. The scale is a 2-point response (“certainly not”/“yes certainly”). The psychometric properties of the measure show good internal consistency; Cronbach alpha=.89 [13].

### Condom Use

To determine condom use, the participants are asked the following question “In the past 2 weeks, thinking about the times you had anal sex with a regular or casual male partner, what percentage of times did you use a condom with someone who was HIV-negative or of unknown status”? This measure showed acceptable psychometric properties [13].

### Acceptability of Condom-Him

The Treatment Acceptability Measure Used for Hiv Prevention Interventions [14] Contains a Set of Items for the Participants to Rate the (1) Appropriateness and Suitability of the Intervention to Assist With Increasing Condom Use, (2) Effectiveness of the Intervention in Helping Them Increase Their Condom Use, and (3) Convenience (ie, Ease of Implementation) and Willingness to Comply With the Intervention Strategies/activities. We Used a 4-Point Response Scale, Which Ranged From “not At All” (0) to “very Much” (4). High Scores Reflect a Positive or Favorable Appraisal of the Intervention. the Psychometric Properties of the Measure Show a Good Internal Consistency; Cronbach Alpha=.86 [15]. in Addition, the Measure Contains Two Qualitative Questions Asking Participants to Indicate “what Makes This Intervention Most and Least Appealing”.

### Feasibility of Condom-Him

The feasibility of the Internet-based intervention will be measured through the recruitment and retention rates, duration of intervention participation, and need for prompts or reminders to complete the post-test questionnaire.

### Utilization of Condom-Him

The utilization of the Internet-based intervention will be measured on the basis of the length of time the participant spent in completing the single session.

### Analysis

#### Preliminary Analysis

Descriptive statistics will be used to characterize the participants in terms of a sociodemographic profile. We will examine the distribution of these variables for normality, which is required for subsequent statistical analyses.

#### Analysis to Address Study Objectives

Independent *t* tests will be used to examine the differences between the control and experimental groups in terms of the participants’ self-efficacy, intention to use condoms, and actual condom use before the intervention and 2 weeks after the intervention.

Descriptive statistics will be used to examine the acceptability rating of the intervention. In addition, qualitative responses of the participants to open-ended questions about what they find most and least appealing about the Condom-Him will be analyzed to identify the strengths and weaknesses of the intervention as perceived by the participants. Recruitment and retention rates for the control and experimental groups will be used to determine the feasibility of the intervention and online data collection methods. Lastly, descriptive statistics will be used to examine the utilization of the intervention.

## Results

The results of this pilot study will enable researchers to understand the feasibility, acceptability, utilization, and preliminary efficacy of the Condom-Him Internet-based intervention in increasing condom use for HIV-positive MSM. In addition, the results of this study will highlight the possibilities and potential challenges inherent in Internet-based intervention research. Further, our results will enable designing of a larger RCT to determine the effectiveness of the Condom-Him Internet-based intervention. Rosser et al [6] showed that in countries with high Internet penetration, Internet-based interventions appear to be the most promising approaches for prevention of HIV infection. The Internet-based intervention could provide a cost-effective and widely accessible strategy for prevention of HIV infection, particularly in HIV-positive MSM, a population that has been under served and yet represents a high-risk group for transmitting HIV through unprotected anal intercourse. Our RCT has been funded, and we have started the recruitment and intervention phase. Results of the pilot RCT are expected in September 2013.

## Discussion

Our proposed study protocol has some limitations. The accuracy of self-reporting may be a cause of concern when dealing with reports of sexual practices. However, previous studies indicate that participants report higher rates of sexual practices when completing Internet-based questionnaires than those of a paper-pencil format. This may be because of the nature of anonymity provided by the Internet-based questionnaires. Further, an Internet-based intervention may perpetuate a divide between those who regularly use a computer and have access to the Internet and those who do not. The recruitment methods proposed in the protocol outline methods that use both online and offline strategies. These strategies aim to minimize the divide in recruiting only those participants that regularly use a computer and the Internet. Participants interested in participating in the study may do complete the Internet-based questionnaire at their convenience. Retention of participants is often a concern with Internet-based interventions. To minimize the loss of participants to follow-up, a friendly email is automatically sent to the participant 2 weeks after the intervention as a reminder to visit the Internet-based intervention to complete the post-test questionnaire. In addition, the participants are notified that if a problem occurs, they are able to contact the research team via email or telephone for help with accessing the Internet-based intervention. Data security is often a concern with Internet-based interventions. Within this study, the data collected are stored on a server, which is located within one of research centers of the affiliated universities. The server is managed by an extensive information technology team with firewalls built into the system. The data are only accessible to the researcher and one information technology expert on the research team. The data collected are coded and downloaded into SPSS for further analysis. Subsequently, the data on the server are removed and destroyed.

## Abbreviations

AIDS: acquired immunodeficiency syndrome
CBIs: computer-technology-based interventions
HIV: human immunodeficiency virus
MSM: men having sex with men
RCT: randomized controlled trial

## Multimedia Appendix 1

CONSORT-EHEALTH checklist V1.6.2 [16].
