# Supplementary material for: An Internet-Based Intervention (Condom-Him) to Increase Condom Use Among HIV-Positive Men Who Have Sex With Men: Protocol for a Randomized Controlled Trial
Source: JMIR Res Protoc. 2013 Oct 13;2(2):e39. doi: 10.2196/resprot.2723 (PMC3806407; doi:10.2196/resprot.2723)
Supplement: Supplementary file 1 [file resprot_v2i2e39_app1.pdf]

CONSORT-EHEALTH Checklist V1.6.2 Report

(based on CONSORT-EHEALTH V1.6), available at [http://tinyurl.com/consort-ehealth-v1-6].

2723

Date completed

5/22/2013 13:08:11

by

Miranda

A randomized control trial of an internet-based intervention to increase condom use among HIV-positive men who are having sex with men: protocol for the Condom-HIM intervention.

TITLE

1a-i) Identify the mode of delivery in the title

"An internet-based intervention"

1a-ii) Non-web-based components or important co-interventions in title

The paper does not mention non-web-based components such as email which is used in the intervention as the emphasis is not on the e-mail component of the intervention.

1a-iii) Primary condition or target group in the title

"Positive men who have sex with men"

ABSTRACT

1b-i) Key features/functionalities/components of the intervention and comparator in the METHODS section of the ABSTRACT

"Participants will be assigned either to use the Condom-HIM intervention (experimental) condition or to view a list of websites containing HIV/AIDS related information (control condition)".

1b-ii) Level of human involvement in the METHODS section of the ABSTRACT

1b-iii) Open vs. closed, web-based (self-assessment) vs. face-to-face assessments in the METHODS section of the ABSTRACT

1b-iv) RESULTS section in abstract must contain use data

"A total of 60 participants, 30 per experimental and 30 per control group will participate in the study".

1b-v) CONCLUSIONS/DISCUSSION in abstract for negative trials

Due to this manuscript being a study protocol, the results are not yet available and therefore conclusions based on the results of the study cannot be discussed at this moment in time.

INTRODUCTION

2a-i) Problem and the type of system/solution

"Many individuals who engage in risky sexual behaviors use the Internet to meet their sexual partners, and the Internet itself may facilitate such risk-taking behaviors [5, 6]. To adhere to current trends, those working in the field of HIV prevention need to incorporate the same medium individuals use in order to reduce risky sexual behaviors. Thus developing technological innovations such as Internet-based interventions is critical".

2a-ii) Scientific background, rationale: What is known about the (type of) system

"Many individuals who engage in risky sexual behaviors use the Internet to meet their sexual partners, and the Internet itself may facilitate such risk-taking behaviors [5, 6]."

METHODS

3a) CONSORT: Description of trial design (such as parallel, factorial) including allocation ratio

"The primary objective of this study is to examine the overall feasibility and acceptability of the Internet-based intervention 2 weeks post intervention as well as examining participants' utilization of the Internet-based intervention".

3b) CONSORT: Important changes to methods after trial commencement (such as eligibility criteria), with reasons

The study is currently being piloted to identify any required changes to the study design.

3b-i) Bug fixes, Downtimes, Content Changes

The study is currently being piloted to identify any required changes to the study design.

4a) CONSORT: Eligibility criteria for participants

"Individuals will be selected to participate in the study if they meet the following preset inclusion criteria: (1) 18 years of age or older, (2) HIV-positive, (3) are men who are having sex with men, (4) engage in unprotected anal intercourse with a partner who is HIV-negative or of unknown status, (5) read English, and (6) have access to a computer and the Internet".

4a-i) Computer / Internet literacy

The study details that participants must have access to a computer and internet. It does not specifically indicate that participants must have computer/internet literacy skills.

4a-ii) Open vs. closed, web-based vs. face-to-face assessments:

"A two-pronged approach will be used to recruit participants. The first method of recruitment will use internet-based methods (i.e. advertisement in chat-rooms, online classified ads, social media such as facebook and twitter, links to the intervention website will be posted on within various HIV/AIDS community center websites). The second method of recruitment will use offline strategies (i.e. flyers and brochures will be posted in various community sexual health clinics). Advertisement in local newspapers will also be used to aid in participant recruitment".

4a-iii) Information giving during recruitment

"Participants interested in the study will be directed to the study website where further information regarding the study will be provided via a video. Participants interested in the study, will be asked to complete an on-line consent form. The consent form will provide information relating to the study in addition to research staff telephone and e-mail contacts for further questions prior to consenting to participate".

4b) CONSORT: Settings and locations where the data were collected

"The data that is collected online will be stored on a secured server located at the Research Chair – Centre Hospitalier de l'Université de Montreal (CR-CHUM)".

4b-i) Report if outcomes were (self-)assessed through online questionnaires

"All measurements are self-administered online".

4b-ii) Report how institutional affiliations are displayed

This information is not addressed within the study protocol, but is in fact identified within the study website. Each of the academic and research institutions are identified in the study website.

5) CONSORT: Describe the interventions for each group with sufficient details to allow replication, including how and when they were actually administered
5-i) Mention names, credential, affiliations of the developers, sponsors, and owners
This information is not identified within the study protocol. The information of the developers is addressed within the actual study website.
5-ii) Describe the history/development process
Information describing the history/development of the intervention is not addressed within this study protocol as the emphasis is on the pilot study itself. A future manuscript will be produced based on the development of the internet-based intervention.
5-iii) Revisions and updating
The study protocol is the first version as it is being pilot tested, there are no revisions to date.
5-iv) Quality assurance methods
This information is not identified within the study protocol. The aim of the study is to determine its feasibility and acceptability. Results from this pilot study will help the research team determine whether or not the current quality measures implemented in the study will need to be revised/addressed.
5-v) Ensure replicability by publishing the source code, and/or providing screenshots/screen-capture video, and/or providing flowcharts of the algorithms used
As the study is currently a pilot study to determine its feasibility, the algorithm has not yet been made available.
5-vi) Digital preservation
The study protocol is being implemented at the moment and therefore this information has not yet been made available as it may be revised based ont he study results.
5-vii) Access
The study protocol is being implemented at the moment and therefore this information is unavailable.
5-viii) Mode of delivery, features/functionalities/components of the intervention and comparator, and the theoretical framework
"The single session is divided into three segments to increase condom use, self-efficacy in condom use and intention to use condoms:
1. Planning condom use when having anal intercourse;
2. Negotiating the use of a condom with a partner; and
3. Choosing not to have sexual intercourse without a condom.
Within each of the three segments of the intervention, participants are given tailored messages pertaining to the focus of the segment. The Intervention Mapping method was used to develop the intervention. This process consists of six consecutive steps to systematically develop health promotion programs using theory, empirical evidence from the literature, and additional evidence from research [12]. The theory-informed methods and practical strategies chosen to increase condom use practices of the target population were supported by Social Cognitive Theory and the Theory of Planned Behavior."
5-ix) Describe use parameters
The study protocol is being implemented at the moment and therefore this information is currently being tested in the pilot study.
5-x) Clarify the level of human involvement
The study protocol is being implemented at the moment and therefore this information is currently being tested in the pilot study.
5-xi) Report any prompts/reminders used
"Participants will be sent an e-mail two-weeks post intervention reminding them to revisit the study website to complete the post-test questionnaires".
5-xii) Describe any co-interventions (incl. training/support)
This information is not relevant as there are no additional co-interventions.
6a) CONSORT: Completely defined pre-specified primary and secondary outcome measures, including how and when they were assessed
"The outcomes of the study will be collected at two points in time: pre-intervention (T0) two-weeks post intervention (T1)".

"The primary outcome measure is condom use. Secondary outcome measures include self-efficacy in condom use, intention to increase condom use, and the acceptability, feasibility, and utilization of Condom-HIM. All measurements are self-administered online".
6a-i) Online questionnaires: describe if they were validated for online use and apply CHERRIES items to describe how the questionnaires were designed/deployed
The information relating to online questionnaire development is not addressed within the study protocol as the study is currently being pilot tested.
6a-ii) Describe whether and how “use” (including intensity of use/dosage) was defined/measured/monitored
"The utilization of the Internet-based intervention will be measured by the length of time participant spent in completing the single session".
6a-iii) Describe whether, how, and when qualitative feedback from participants was obtained
"participants’ qualitative responses to open questions in regards to the acceptability of Condom-HIM will be content analyzed to identify any problems with the intervention".
6b) CONSORT: Any changes to trial outcomes after the trial commenced, with reasons
This information is not relevant at this point in time as the study is currently being tested.
7a) CONSORT: How sample size was determined
7a-i) Describe whether and how expected attrition was taken into account when calculating the sample size
"The sample size calculation is based on the rule of 10 cases per independent variable included in the analysis".
7b) CONSORT: When applicable, explanation of any interim analyses and stopping guidelines
This information is not relevant at this point in time as the study is currently being tested.
8a) CONSORT: Method used to generate the random allocation sequence
"The participant assignment protocol will be based on a ratio of 1:1, which will be performed by a computer program".
8b) CONSORT: Type of randomisation; details of any restriction (such as blocking and block size)
This information is not relevant to the study as the randomization allocation sequence is a 1:1 ratio.
9) CONSORT: Mechanism used to implement the random allocation sequence (such as sequentially numbered containers), describing any steps taken to conceal the sequence until interventions were assigned
"The computer program will automatically allocate participants at random to either the experimental or control condition".
10) CONSORT: Who generated the random allocation sequence, who enrolled participants, and who assigned participants to interventions
"Due to the automatic nature of the allocation process, there are no direct exposure to the treatment allocation process by any members of the research team".
11a) CONSORT: Blinding - If done, who was blinded after assignment to interventions (for example, participants, care providers, those assessing outcomes) and how
11a-i) Specify who was blinded, and who wasn’t
The researcher will be blinded to the participant’s treatment assignment. Only the research assistant, who will be downloading the data, will be the only individual aware of the participant’s assignment. The research assistant will not have any participation in the random assignment of participants as this process has been removed from any individuals and is completely being conducted by the computer system programming.

11a-ii) Discuss e.g., whether participants knew which intervention was the "intervention of interest" and which one was the "comparator"
Participants in the study will not be blinded to their assigned arm as this is not possible. Individuals within the control arm receiving a list of various website will be aware they have not been allocated to the tailored online intervention. Participants in the experimental arm (Condom-HIM) will be aware that they have been allocated to tailored online intervention.
11b) CONSORT: If relevant, description of the similarity of interventions
This information is not relevant as their is no placebo intervention but rather a control condition consisting of a list of websites.
12a) CONSORT: Statistical methods used to compare groups for primary and secondary outcomes
Independent t-tests will be used to examine the differences between the control and experimental groups in terms of participants' self-efficacy, intention to use condoms, and actual condom use prior to the intervention and 2 weeks post intervention.
"Descriptive statistics will be used to examine the intervention's acceptability rating. In addition, participants' qualitative responses to open questions in regards to the acceptability of Condom-HIM will be content analyzed to identify any problems with the intervention. Recruitment and retention rates for both the control and experimental groups will be used to determine the feasibility of the intervention and online data collection methods. Lastly, descriptive statistics will be used to examine utilization of the intervention".
12a-i) Imputation techniques to deal with attrition / missing values
"The feasibility of the Internet-based intervention will be measured through the recruitment and retention rates, duration of intervention participation, and need for prompts or reminders to complete the post-test questionnaire".
12b) CONSORT: Methods for additional analyses, such as subgroup analyses and adjusted analyses
This information is not relevant as the study is based on the feasibility and acceptability of the internet-based intervention.

RESULTS

13a) CONSORT: For each group, the numbers of participants who were randomly assigned, received intended treatment, and were analysed for the primary outcome
This information relating to study results is not available as this manuscript is being submitted as a study protocol.
13b) CONSORT: For each group, losses and exclusions after randomisation, together with reasons
This information relating to study results is not available as this manuscript is being submitted as a study protocol.
13b-i) Attrition diagram
This information relating to study results is not available as this manuscript is being submitted as a study protocol.
14a) CONSORT: Dates defining the periods of recruitment and follow-up
This information relating to study results is not available as this manuscript is being submitted as a study protocol.
14a-i) Indicate if critical "secular events" fell into the study period
This information relating to study results is not available as this manuscript is being submitted as a study protocol.
14b) CONSORT: Why the trial ended or was stopped (early)
This information relating to study results is not available as this manuscript is being submitted as a study protocol.
15) CONSORT: A table showing baseline demographic and clinical characteristics for each group
This information relating to study results is not available as this manuscript is being submitted as a study protocol.
15-i) Report demographics associated with digital divide issues
This information relating to study results is not available as this manuscript is being submitted as a study protocol.
16a) CONSORT: For each group, number of participants (denominator) included in each analysis and whether the analysis was by original assigned groups
16-i) Report multiple "denominators" and provide definitions
This information relating to study results is not available as this manuscript is being submitted as a study protocol.
16-ii) Primary analysis should be intent-to-treat
This information relating to study results is not available as this manuscript is being submitted as a study protocol.
17a) CONSORT: For each primary and secondary outcome, results for each group, and the estimated effect size and its precision (such as 95% confidence interval)
This information relating to study results is not available as this manuscript is being submitted as a study protocol.
17a-i) Presentation of process outcomes such as metrics of use and intensity of use
This information relating to study results is not available as this manuscript is being submitted as a study protocol.
17b) CONSORT: For binary outcomes, presentation of both absolute and relative effect sizes is recommended
This information relating to study results is not available as this manuscript is being submitted as a study protocol.
18) CONSORT: Results of any other analyses performed, including subgroup analyses and adjusted analyses, distinguishing pre-specified from exploratory
This information relating to study results is not available as this manuscript is being submitted as a study protocol.
18-i) Subgroup analysis of comparing only users
This information relating to study results is not available as this manuscript is being submitted as a study protocol.
19) CONSORT: All important harms or unintended effects in each group
This information relating to study results is not available as this manuscript is being submitted as a study protocol.
19-i) Include privacy breaches, technical problems
This information relating to study results is not available as this manuscript is being submitted as a study protocol.
19-ii) Include qualitative feedback from participants or observations from staff/researchers
This information relating to study results is not available as this manuscript is being submitted as a study protocol.

DISCUSSION

20) CONSORT: Trial limitations, addressing sources of potential bias, imprecision, multiplicity of analyses
20-i) Typical limitations in ehealth trials
This information relating to study discussion based on results of the study is not available as this manuscript is being submitted as a study protocol. Although limitations of the proposed study protocol are outlined within the discussion section.

"Self-reporting accuracy is of concern when dealing with reports of sexual practices. Although as identified previously, evidence demonstrates that participants report higher rates of sexual practices when completing Internet based questionnaires than paper-pencil format".

21) CONSORT: Generalisability (external validity, applicability) of the trial findings
21-i) Generalizability to other populations
This information relating to study discussion based on results of the study is not available as this manuscript is being submitted as a study protocol.
21-ii) Discuss if there were elements in the RCT that would be different in a routine application setting

This information relating to study discussion based on results of the study is not available as this manuscript is being submitted as a study protocol.

22) CONSORT: Interpretation consistent with results, balancing benefits and harms, and considering other relevant evidence

22-i) Restate study questions and summarize the answers suggested by the data, starting with primary outcomes and process outcomes (use)

This information relating to study discussion based on results of the study is not available as this manuscript is being submitted as a study protocol.

22-ii) Highlight unanswered new questions, suggest future research

This information relating to study discussion based on results of the study is not available as this manuscript is being submitted as a study protocol.

Other information

23) CONSORT: Registration number and name of trial registry

"The study is registered with the ClinicalTrials.gov (NCT01726153)"

24) CONSORT: Where the full trial protocol can be accessed, if available

The full trial protocol is not available elsewhere.

25) CONSORT: Sources of funding and other support (such as supply of drugs), role of funders

"The authors would like to thank Fonds de la Recherché en Santé du Québec for funding granted towards the development of the Condom-HIM Internet-based application, and the Québec Interuniversity Nursing Intervention Research Group for funding provided for the pilot RCT".

X26-i) Comment on ethics committee approval

"The study has gained REB approval from both the academic and research institutions".

x26-ii) Outline informed consent procedures

"Participants interested in the study, will be asked to complete an on-line consent form. The consent form will provide information relating to the study in addition to research staff telephone and e-mail contacts for further questions prior to consenting to participate".

X26-iii) Safety and security procedures

"The data that is collected online will be stored on a secured server located at the Research Chair – Centre Hospitalier de l'Université de Montreal (CR-CHUM)".

X27-i) State the relation of the study team towards the system being evaluated

"The authors declare that they have no competing interest"
